# Vaccination Ascribed as the Cause of a Death from Lockjaw

**Published:** 1894-02

**Authors:** 


ARTICLE IV.

### VACCINATION ASCRIBED AS THE CAUSE OF A DEATH FROM LOCKJAW.

Marjorie Woodruff, of Patchogue, Va., the five-year-old daughter of Charles Woodruff, a well-to-do butcher of Bellport, died on Tuesday morning last of lockjaw. Four weeks ago to-day she was vaccinated by Dr. S. A. Toms, of Bellport. The first indication that anything was wrong came on Thanksgiving day. At dinner Mr. Woodruff noticed that his daughter was crying.

"What's the matter?" he inquired.

"My mouth hurts me," said the child.

"Nonsense," said Mr. Woodruff, "Go on and eat your dinner."

The child tried in vain to eat a piece of turkey, and finally left the table in tears. At breakfast the next morning Marjorie again complained that she could not eat. Mr. Woodruff noticed that she had great difficulty in working her jaws. She complained of a sore tongue, and an examination revealed the presence of canker sores. Mr. Woodruff made up his mind that these had caused the trouble and thought no more of the matter until Sunday, when the child complained of a sore throat. Her tongue was worse than it had been, and she was unable to open her jaws more than half an inch.

On Sunday night she had a spasm, and Dr. Toms was sent for. He saw that she was suffering from an ulcerated sore throat and a sore tongue. The spasm and the inability to work the jaws pointed to lockjaw, and he made a careful examination of the girl to discover if she had been cut or wounded in any way. The examination revealed nothing. The only wound found was the vaccination mark, and that was almost healed. Greatly puzzled, Dr. Toms treated the child for sore throat, and kept a close watch for further symptoms of lockjaw.

He succeeded, in a measure, in curing the throat trouble, but on Monday the child had another spasm. Her jaw closed tight, and it was impossible to pry them apart. Dr. Toms decided that the child had lockjaw, and began treating her for that trouble. The attack was a very light one, and on Monday night Dr. Toms assured Mr. Woodruff that the child would live.

At 11 o'clock on Monday night, about two hours after the doctor had left, Marjorie had another spasm. Dr. Toms got her mouth open, and, with a spoon, managed to get a good luck at her throat. He saw that it was badly ulcerated. While he was examing the child her jaws closed with a snap on the spoon, and all effort to make her release her hold proved futile. It was not until she had been put under the influence of an anæsthetic that she opened her mouth.

The next day he had a consultation with Dr. A. H. Terry, of Patchogue. The two physicians decided that the girl had had a slight attack of lockjaw, but would recover. They also discovered that œdema of the lungs had set in. Dr. Terry remained only a short time after the consultation, but Dr. Toms stayed until 11 o'clock, when the child died. The death was a complete surprise.

To a reporter Mr. Woodruff said: "I have been told that lockjaw, as the result of vaccination, is something unheard of; yet if the trouble didn't come from that what did it come from? The child hadn't another cut or bruise on her entire body. The vaccination was the only mark on her, and that had almost healed. She had a couple of decayed teeth, but all children are troubled that way more or less, and no one seems to think that that could have caused anything so serious as lockjaw. The child suffered frightfully and had spasm after spasm. When she was in a spasm, her jaws were as tight as a vise, and it was utterly impossible to get them apart. Even when she was perfectly conscious she could not get them more than half an inch apart.

"Some people laugh at the idea of it being the vaccination that caused the lockjaw, but say for me that no member of my family, not even my dog there, will ever be vaccinated again as long as I live. I believe it caused my little one's death, and will never give it a chance to carry off another one of my family."

Dr. Toms said: "The child certainly had lockjaw. There was no doubt about that. It is one of the most mystifying cases that has ever come under my observation, and I am doing everything I can to find out how the child contracted the trouble. There is a great deal of talk about vaccination having brought on the lockjaw. In all my practice I never heard of vaccination resulting in that way, and physicians whom I have consulted say the same thing. I have written to the Surgeon-General of the United States army to find out if he ever heard of such a case, and also to the vaccine farm.

"Now, although this child had lockjaw, that was not the sole cause of death. We had the lockjaw under control and could have cured the child had she had any vitality. She hadn't, however, and œdema of the lungs set in and she died.

"Now, as to the cause of the lockjaw. The disease is caused by a germ entering the system. It can only enter through a cut. The disease always becomes apparent in from 7 to 12 days. This child was vaccinated four weeks ago to-day. It was over three weeks, in fact, nearly 24 days, before the disease showed in the child. That would certainly indicate that it did not come from the operation of vaccination. It is not impossible that the lockjaw came from the vaccination wound. If a germ entered the wound it would cause lockjaw as quickly as if it entered through an ordinary cut. The only way the germ could have gotten in was through the dressing. The child's parents dressed the wound. I am sure they exercised the utmost care.

"Another way in which lockjaw may be contracted is through the mouth, if the membrane is broken. In the case of this child there was a tongue covered with canker sores and an ulcerated sore throat. It seems to me that the germ entered through the mouth. It is a most remarkable case all through, and one which is bound to attract the attention of medical men all over the country."

Dr. Terry agrees with Dr. Toms about the case. The people about Patchogue are greatly excited over the case. Almost everyone has submitted to vaccination recently, and many are beginning to worry. Those who have not been vaccinated absolutely refuse to submit to the operation.

Dr. Edson, of the health department, said: The slightest abrasion may lead to lockjaw if the germ of the disease enters the system through the wound. We are not sure yet that it is a germ disease, but believe that it is. Vaccination alone could not cause it. We have vaccinated over 200,000 people, and never has a case of lockjaw resulted.

"In certain localities, for instance, at the other end of Long Island, about Sag Harbor, there are many cases of lockjaw, and it is dangerous to get the slightest scratch."

